# Prediction of individualized therapeutic vulnerabilities in cancer from genomic profiles

**DOI:** 10.1093/bioinformatics/btu164

**Published:** 2014-03-24

**Authors:** Bülent Arman Aksoy, Emek Demir, Özgün Babur, Weiqing Wang, Xiaohong Jing, Nikolaus Schultz, Chris Sander

**Affiliations:** ^1^Computational Biology Center, Memorial Sloan-Kettering Cancer Center, New York, NY 10065 and ^2^Tri-Institutional Training Program in Computational Biology & Medicine, New York, NY 10065, USA

## Abstract

**Motivation:** Somatic homozygous deletions of chromosomal regions in cancer, while not necessarily oncogenic, may lead to therapeutic vulnerabilities specific to cancer cells compared with normal cells. A recently reported example is the loss of one of the two isoenzymes in glioblastoma cancer cells such that the use of a specific inhibitor selectively inhibited growth of the cancer cells, which had become fully dependent on the second isoenzyme. We have now made use of the unprecedented conjunction of large-scale cancer genomics profiling of tumor samples in The Cancer Genome Atlas (TCGA) and of tumor-derived cell lines in the Cancer Cell Line Encyclopedia, as well as the availability of integrated pathway information systems, such as Pathway Commons, to systematically search for a comprehensive set of such epistatic vulnerabilities.

**Results:** Based on homozygous deletions affecting metabolic enzymes in 16 TCGA cancer studies and 972 cancer cell lines, we identified 4104 candidate metabolic vulnerabilities present in 1019 tumor samples and 482 cell lines. Up to 44% of these vulnerabilities can be targeted with at least one Food and Drug Administration-approved drug. We suggest focused experiments to test these vulnerabilities and clinical trials based on personalized genomic profiles of those that pass preclinical filters. We conclude that genomic profiling will in the future provide a promising basis for network pharmacology of epistatic vulnerabilities as a promising therapeutic strategy.

**Availability and implementation**: A web-based tool for exploring all vulnerabilities and their details is available at http://cbio.mskcc.org/cancergenomics/statius/ along with supplemental data files.

**Contact**: statius@cbio.mskcc.org

**Supplementary information:** Supplementary data are available at *Bioinformatics* online.

## 1 INTRODUCTION

**Fig. 1.**
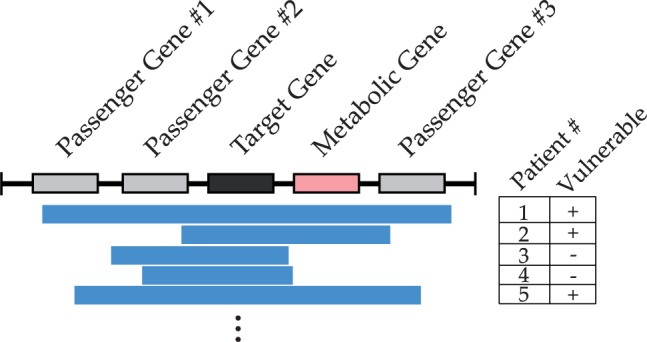
Deletions often result in the loss of a locus (horizontal bars) that often contains multiple genes. These deletions can sometimes cause loss of a metabolic gene as a passenger event. This type of alterations are not lethal to a cell if another gene can sufficiently carry the load of the deleted metabolic gene, but the loss of these passenger genes may create therapeutic vulnerabilities in tumors

Comprehensive cancer profiling studies, such as The Cancer Genome Atlas (TCGA) and other studies by the International Cancer Genome Consortium, have helped identify many genomic alterations in cancer genomes, including homozygous deletions that often result from genomic instability. Deletions that confer a proliferative advantage, such as the homozygous deletion of a tumor-suppressor gene, are selected in cancer cells via clonal expansion (Hanahan and Weinberg, 2011). Other deletions with relatively little effect on the tumor’s proliferative capabilities can be seen at low frequencies when they are, by chance, co-selected with other oncogenic events. Both types of deletions, however, result in the loss of a locus that often contains multiple genes. Such a deletion may not be lethal to a cell if one or more unaffected partner genes (e.g. an isoenzyme) can sufficiently carry the load of the deleted partner, but the loss of these passenger genes may create therapeutic vulnerabilities (Fig. 1). On loss of an initial gene, interference with the function of its partner gene(s) may result in cell death, a phenomenon known as synthetic lethality.

Muller *et al.* (2012) recently published a case study for synthetic lethality for glioblastoma. Enolase performs an essential function in cells, catalyzing the conversion of 2-phosphoglycerate and phosphoenolpyruvate in the glycolytic pathway. At least three known genes encode enolase isoenzymes: ENO1, ENO2 and ENO3. ENO1 has been shown to be homozygously deleted in certain glioblastomas, probably as a passenger event to the deletion of ERFFI1, but the tumor cells are able to survive because of the activity of other enolase encoding genes, in particular ENO2. Although the loss of ENO1 alone may not be lethal, cancer cells lacking ENO1 are selectively vulnerable to the loss of ENO2 (i.e. synthetic lethality), whereas non-cancer cells with intact ENO1 can tolerate a loss of ENO2.

Most of the cancer genomics research focuses on identifying driver alterations by frequency or occurrence pattern and exploiting them to treat cancer (Ciriello *et al.*, 2012; Kim *et al.*, 2013; Mermel *et al.*, 2011; Taylor *et al.*, 2008). However, there is an opportunity to exploit synthetic lethalities specific to particular populations of cancer cells created by the homozygous loss of genes responsible for core cellular functions. These are rare patient-specific events, and there are no existing tools for identifying these vulnerabilities for a given patient. A system that can efficiently analyze genomic data from biological samples to identify particular therapeutic vulnerabilities in cancer cells specific to those samples based on potential synthetic lethal partner genes can identify personalized treatments to inhibit or kill those cancer cells.

**Fig. 2.**
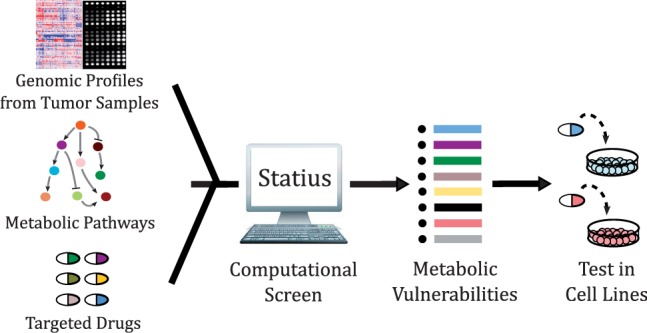
Overall process of identification of therapeutic vulnerabilities. Statius imports cancer genomics data provided by the cBioPortal (Cerami *et al.*, 2012; Gao *et al.*, 2013), along with pathway and drug annotations from a customizable list of external resources. It then produces a list of sample-specific vulnerabilities categorized by the cancer study as output. These potential vulnerabilities can be further tested in cell lines bearing the vulnerability of interest

Here, we describe a computational method, Statius (named after the Roman poet, Publius Papinius Statius, who is known for his famous poems *Achilleid* and *Thebaid*), to systematically predict metabolic vulnerabilities in tumor samples from genomic profiles. We present results obtained from the analysis of 16 publicly available cancer studies (Fig. 2). Integrating data, in an automated manner, from multiple data resources—including several pathway databases, drug–target annotation resources and cancer genomics utilities—we were able to predict sample-specific metabolic vulnerabilities, which result from a homozygous deletion event in the corresponding sample, and list drugs that can help exploit each particular vulnerability. The complete list of the predicted vulnerabilities can be found at http://cbio.mskcc.org/cancergenomics/statius.

## 2 RESULTS

### 2.1 Data collection

#### 2.1.1 Drug–target relationships

As a first step in our analysis, we collected information on available targeted drugs and their known targets. For this, we gathered drug–target data from multiple curated data resources including, but not limited to, DrugBank (Knox *et al.*, 2011) and KEGG Drug (Kanehisa *et al.*, 2012) using the PiHelper tool (Aksoy *et al.*, 2013). We further collected information from the National Cancer Institutes’ Online Cancer Resource (http://cancer.gov) to annotate whether a drug has been approved for cancer therapy. We were able to extract information for 7817 targeted drugs and 17 981 drug–target relationships corresponding to these drugs. To remove non-specific drugs, we excluded from our initial analysis drugs that have more than five known targets, leaving 7625 drugs and 15 210 drug targets covering 1674 genes.

#### 2.1.2 Gene sets representing isoenzymes

We next created a list of all known metabolic isoenzymes as representatives of synthetic lethal gene groups. To accomplish this, we used curated human metabolic pathway information from Pathway Commons in BioPAX format (Cerami *et al.*, 2011; Demir *et al.*, 2010). We specifically collected metabolism pathways provided by Reactome and HumanCyc databases (Croft *et al.*, 2011; Romero *et al.*, 2005). Using these data resources, we extracted official gene symbols from protein entities that catalyze the same metabolic reaction and considered them as isoenzymes.

In addition to these pathway databases, we also used metabolic enzyme information provided by the KEGG Enzyme database (Kanehisa *et al.*, 2012). For each enzyme, identified by a specific Enzyme Commission (EC) number, we extracted the corresponding human gene symbols and grouped them as isoenzyme gene sets.

Combining data from these three resources, we were able to extract 1290 unique gene sets. We filtered out 1063 gene sets consisting of more than five genes, as our preliminary screen showed that gene sets with more than five genes do not increase the number of predicted vulnerabilities in a considerable manner, as well as those that consist of only non-targetable genes.

#### 2.1.3 Cancer studies and genomic profiles

Next, we obtained genomic profiles, minimally somatic copy-number alteration (CNA) data, from publicly available cancer studies. To obtain information on multiple studies, we used the web service of the cBioPortal for Cancer Genomics (Cerami *et al.*, 2012; Gao *et al.*, 2013). We used categorical CNA information to identify whether a gene is homozygously deleted for a given sample. Whenever available, we also collected normalized gene expression levels for a homozygously deleted gene of interest to see whether the gene is underexpressed compared with the rest of the samples in the same cancer study. For this analysis, we used genomic profiles for 5971 samples (4999 tumor samples and 972 cell lines) from 16 different cancer studies that had publicly available CNA data (Table 1). All but two studies we included in our set had also the mRNA expression data available.

**Table 1.** We screened 5971 samples from 16 different cancer studies

Cancer study	Genomic profiles				
	Source	Samples	CNA	Exp.	Tissue
Acute myeloid leukemia	TCGA (The Cancer and Genome Atlas, 2013)	191	+	+	Bone marrow
Adenoid cystic carcinoma	MSKCC (Ho *et al.*, 2013)	60	+	−	−
Bladder cancer	MSKCC (Iyer *et al.*, 2013)	97	+	+	Bladder
Breast invasive carcinoma	TCGA (Koboldt *et al.*, 2012)	913	+	+	−
CCLE	Novartis/broad (Barretina *et al.*, 2012)	972	+	+	−
Colon and rectum adenocarcinoma	TCGA (Muzny *et al.*, 2012)	575	+	+	Colon
Glioblastoma multiforme	TCGA (The Cancer and Genome Atlas, 2008)	497	+	+	Brain
Head and neck squamous cell carcinoma	TCGA	306	+	+	−
Kidney renal clear cell carcinoma	TCGA (Creighton *et al.*, 2013)	436	+	+	−
Lung adenocarcinoma	Broad (Imielinski *et al.*, 2012)	182	+	−	Lung
Lung adenocarcinoma	TCGA	230	+	+	Lung
Lung squamous cell carcinoma	TCGA (Hammerman *et al.*, 2012)	197	+	+	Lung
Ovarian serous cystadenocarcinoma	TCGA (The Cancer and Genome Atlas, 2011)	569	+	+	Ovary
Prostate adenocarcinoma	MSKCC (Taylor *et al.*, 2010)	194	+	+	Prostate
Sarcoma	MSKCC/broad (Barretina *et al.*, 2010)	207	+	+	Soft tissue
Uterine corpus endometrioid carcinoma	TCGA (Kandoth *et al.*, 2013)	363	+	+	Uterus
	**Total**	5971			

*Note*: The majority of the cancer studies were from TCGA, and the others were from different individual institutions. We annotated each cancer study with its tissue of origin in accordance with the TiGER database (Liu *et al.*, 2008). TCGA: The Cancer Genome Atlas; MSKCC: Memorial Sloan-Kettering Cancer Center; Broad: Broad Institute; CNA: DNA copy-number alteration; Exp: mRNA expression; −: tissue annotation not available.

#### 2.1.4 Additional gene annotations

Most of the isoenzymes show tissue-specific expression patterns where the expression of an isoenzyme is restricted to a single or multiple tissues. We wanted to use this context-specific background information in our analysis and take the tissue associated with a cancer study, when trying to find vulnerabilities. It is also known that some genes are essential for the viability of a cell, and therefore, targeting such a gene causes some level of toxicity to all cells in a non-selective manner, making these genes unpreferred targets for an ideal therapy.

Therefore, we annotated the genes to recognize tissue-specific expression patterns and also essentiality. Using Tissue-specific Gene Expression and Regulation (TiGER) database, we first extracted tissue-specific genes. We also, when possible, annotated the cancer studies with a tissue in accordance with the TiGER terminology (Liu *et al.*, 2008). These data allowed us to query for a given sample, associated with a cancer study and thus a tissue, whether a gene of interest is expected to be expressed. We next used data provided by Database of Essential Genes (DEG) to annotate whether a gene of interest is essential for the organism (Zhang *et al.*, 2004). Using this dataset, we mark a human gene as essential if its homologue in any of the well-known model organisms is known to be essential for the viability of that particular organism.

### 2.2 Identification of vulnerabilities

#### 2.2.1 Sample-specific vulnerabilities

Putting all these information together, we then analyzed each sample in our dataset—in the context of the cancer study it belongs to—to identify potential metabolic vulnerabilities. To accomplish this, for a given cancer study, a tumor or cell line sample and an isoenzyme gene set, we looked for cases where (i) one or more isoenzymes are lost because of homozygous deletion, (ii) and the other expressed isoenzymes can be selectively targeted by at least one drug. Once we found the vulnerabilities in this selective manner, we also included all possible drugs, selective or not, in our final results.

#### 2.2.2 Vulnerability scores

To sort all predicted vulnerabilities based on their internal consistency and annotations, we assigned a score over 4.0 to each sample-specific vulnerability. For this, we checked whether a given sample-specific vulnerability satisfied any of the following criteria: (i) the homozygously deleted gene is also underexpressed (or not expressed), (ii) there are any Food and Drug Administration (FDA)-approved drugs in the suggested drug list, (iii) there are any ‘cancer’ drugs in the suggested drug list, where a cancer drug means a drug that is currently FDA-approved and being used in cancer treatment and (iv) the target of the suggested drug is not an essential gene in any of the model organisms.

#### 2.2.3 Vulnerabilities in tumor samples and matching cell lines

**Fig. 3.**
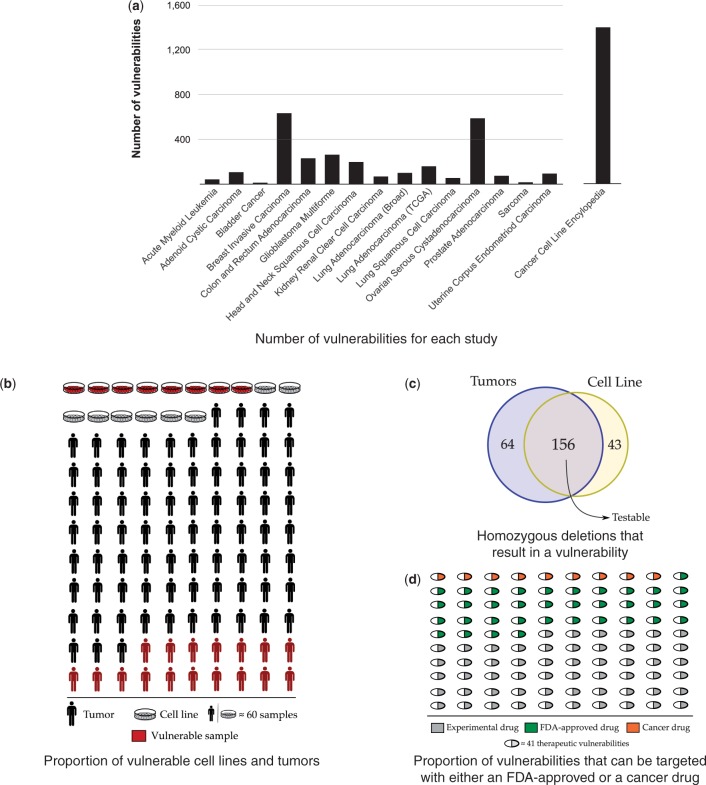
Systematic screening of cancer samples revealed metabolic vulnerabilities that are of therapeutic interest in a uniform way across different cancer types. (**a**) Across 16 cancer studies, we identified 4101 vulnerabilities. (**b**) We screened 5971 samples (972 cell lines and 4999 tumor samples) and found 1019 tumor samples and 482 cancer cell lines to have possible metabolic vulnerabilities (red). (**c**) All vulnerabilities were attributable to 263 distinct homozygous deletion events; 156 (60%) of these deletions were shared between at least one cell line and one tumor sample. (**d**) Forty-four percent of all identified vulnerabilities can potentially be targeted with an FDA-approved drug (green) and furthermore 8% with an FDA-approved drug that is currently known to be used in cancer therapy (orange)

We ran our analysis on 5971 cancer samples covering 16 distinct cancer studies and identified 4104 metabolic vulnerabilities in 1019 tumor samples and 482 cancer cell lines (Figs 3a and b). In all, 146 of 4104 (4%) vulnerabilities had a score of 3, whereas 31, 51 and 14% vulnerabilities had a score of 2, 1 and 0, respectively. Overall, we were able to identify 263 distinct homozygous deletions that cause a predicted vulnerability (Table 2; Supplementary Data for complete results); we found that 220 of 263 homozygous deletions were present in tumor samples and 71% of these had at least one matching cell line (Fig. 3c). We also found that 1833 (44%) of the vulnerabilities can potentially be targeted with at least one FDA-approved drug, but in a less-selective manner (Fig. 3d). One such example to this less-selective targeting is the potential use of methotrexate when either DHFR or DHFRL1 is deleted in the sample, although the drug targets both genes in this isoenzyme pair (Table 3). Furthermore, we found that in 1695 of 4104 (41%) vulnerabilities that we identified, intervention with drugs will involve targeting at least one essential enzyme (Supplementary Fig. S1).

**Table 2.** The 20 most common candidate therapeutic vulnerabilities detected in the analysis of 5971 cancer samples from 16 different studies

Serial number	Isoenzyme set	Deleted gene	Vulnerable samples		Metabolic reaction	Drugs
			Tumors	Cell lines		
1	EXTL2, EXTL3	EXTL3	173	47	Glucuronyl-galactosyl-proteoglycan 4-alpha-N-acetylglucosaminyltransferase	Uridine-diphosphate-N-acetylglucosamine
2	PAPSS1, PAPSS2	PAPSS2	97	17	Adenylyl-sulfate kinase	Adenosine-5′-phosphosulfate
3	CPT1C, CPT1B, CPT2, CPT1A	CPT1B	90	10	Carnitine O-palmitoyltransferase	L-carnitine
4	A2M, BMP1	BMP1	68	2	High-density lipoprotein–mediated lipid transport	Becaplermin
5	GOT1, GOT2, GOT1L1	GOT1L1	65	27	Aspartate degradation II	Maleic acid, 4′-deoxy-4′-acetylyamino-pyridoxal-5′-phosphate
6	GYG1, GYG2	GYG2	58	0	Glycogenin glucosyltransferase	UDP-D-galactose
7	ATP2C1, ATP2C2	ATP2C2	57	20	Calcium transport I	Desflurane/halothane
8	ADA, ADAT3	ADAT3	53	13	Adenine and adenosine salvage III	Pentostatin
9	SAT1, SAT2	SAT2	48	44	Diamine N-acetyltransferase	Diminazene
10	FNTA, PGGT1B	PGGT1B	47	15	Protein geranylgeranyltransferase type I	Tipifarnib
11	DHFR, DHFRL1	DHFR	47	5	Dihydrofolate reductase	5-chloryl-2,4,6-quinazolinetriamine
12	AKR1B10, AKR1B1, CYP2E1	CYP2E1	42	33	Methylglyoxal degradation III	Tolrestat
13	TK1, TK2	TK2	42	8	Thymidine kinase	Dithioerythritol
14	ACAT1, ACAT2	ACAT2	39	23	Acetyl-CoA C-acetyltransferase	Sulfasalazine
15	ENO1, ENO2, ENO3	ENO1	37	18	Phosphopyruvate hydratase	2-phosphoglycolic acid
16	ACAT1, ACAT2	ACAT1	36	22	Acetyl-CoA C-acetyltransferase	Pyripyropene A
17	MTHFD1, MTHFD1L	MTHFD1L	34	24	Formate—tetrahydrofolate ligase	LY374571/LY249543
18	ALDH2, ALDH3A2	ALDH3A2	30	28	Putrescine degradation III	Daidzin
19	TRYP1, CAT	TYRP1	12	71	Ethanol degradation IV	Fomepizole
20	AMY1A/B/C, AMY2A, AMY2B	AMY1A/B/C	1	61	Alpha-amylase	Acarbose

*Note*: Our analysis revealed 263 candidate vulnerabilities. Each of these vulnerabilities is associated with a gene set that represents isoenzymes that catalyze a metabolic reaction, and deletion of one or more partner genes results in a vulnerability if there are targeted drug(s) that can selectively inhibit the other enzymes in the gene set. The majority of the vulnerabilities in tumors were also present in at least one cell line (see Supplementary Table S1 for an extended version of this table).

**Table 3.** Vulnerabilities that can potentially be exploited with a cancer drug—a drug that is approved by FDA for use in cancer therapy

Serial number	Isoenzyme set	Cases	Metabolic reaction	Drug(s) of interest
1	TOP2B*, TOP2A*	70	DNA topoisomerase (ATP)-hydrolysing	Daunorubicin, Epirubicin, Doxorubicin, Etoposide, Dexrazoxane
2	DHFR*, DHFRL1*	68	dihydrofolate reductase	Methotrexate, Pemetrexed, Pralatrexate
3	IKBKE*, TBK1*, IKBKB, CHUK*	46	IkappaB kinase	Arsenic trioxide
4	LIG1, LIG3, LIG4*	43	DNA ligase (ATP)	Bleomycin
5	P4HB*, MTTP*	34	Chylomicron-mediated lipid transport	Vandetanib, Nilotinib, Imatinib, Bosutinib, Dasatinib
6	RRM1*, RRM2*	33	Synthesis and interconversion of nucleotide di- and triphosphates	Clofarabine, Fludarabine, Gemcitabine
7	CMPK1, CMPK2*	20	Deoxycytidylate kinase	Gemcitabine
8	GGPS1*, FDPS*	7	Dimethylallyltranstransferase	Zoledronate
9	PTGS2, PTGS1*	3	Taglandin-endoperoxide synthase	Thalidomide, Lenalidomide
10	TXNRD1, TXNRD2*, TXNRD3	5	Thioredoxin-disulfide reductase	Arsenic trioxide
11	TOP1, TOP3A*, TOP1MT, TOP3B	4	Irinotecan	Topotecan

*Note*: In some cases, deletion of either of partner genes can result in a therapeutic vulnerability. For example, TOP2A and TOP2B are isoenzymes that function as ATP-hydrolyzing DNA topoisomerases. Of 5971 cases (tumor or cell line samples), 70 have either TOP2B- or TOP2A-deletion (*). Either of these deletions creates vulnerabilities that can be exploited with drugs, such as Doxorubicin or Etoposide, that selectively inhibit these isoenzymes.

**Fig. 4.**
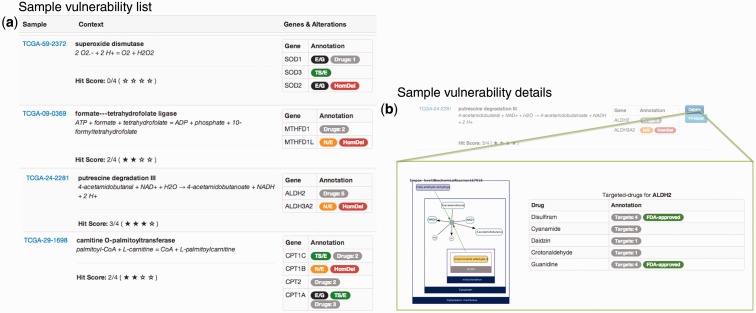
Four vulnerabilities, with different contexts, identified in the ovarian serous cystadenocarcinoma (TCGA) cancer study. Each vulnerability is associated with a sample and a metabolic context. Furthermore, for each vulnerability, the gene sets are annotated to provide information whether a gene is homozygously deleted (red; HomDel), essential (black; E/G), not expressed (orange; N/E), shows tissue-specific expression (green; TS/E) or is known to be selectively targeted by a drug (gray; drugs: N). For gene sets extracted from Pathway Commons, the metabolic reaction of interest is visualized as an image that was produced by ChiBE (Babur *et al.*, 2010)

To allow better investigation of these vulnerability results, we developed a web user interface accessible at http://cbio.mskcc.org/cancergenomics/statius. The interface allows users to browse vulnerabilities either through a cancer study or gene set-based views, and for each predicted vulnerability, it provides additional context annotations and information with external links (Fig. 4).

## 3 METHODS

### 3.1 Obtaining information on isoenzymes

#### 3.1.1 From pathway resources: Reactome and HumanCyc

We obtained biological pathway information from both Reactome and HumanCyc (Croft *et al.*, 2011; Romero *et al.*, 2005). We used entity-level normalized BioPAX Level 3 outputs for both data resources. The normalization was accomplished through Pathway Commons 2 and cPath 2 software to standardize external references of entities in these pathway datasets (Cerami *et al.*, 2006). We then parsed these BioPAX Level 3 pathway data using Paxtools library and extracted isoenzyme gene sets using the following procedure [(Demir *et al.*, 2010, 2013); http://biopax.org/paxtools.php]: we first iterated for all *BiochemicalReaction*s that have at least one *Controller* to it. For a given *BiochemicalReaction*, we then iterated for all *Controller* entities of the reaction and obtained corresponding *Xref*s (external references). Using *Xref*s that map an entity to HGNC (HUGO Gene Nomenclature Committee), we collect HGNC gene symbols of corresponding controllers and treat them as isoenzyme groups. For each isoenzyme group, we keep the name of the reaction, the pathway it belongs to and an image of the corresponding reaction associated with that particular group for later visualization features. All reaction images were generated with ChiBE (Babur *et al.*, 2010). For the described procedure, we used the whole HumanCyc dataset, but for Reactome, we only used the reactions that belong to the *Metabolism* pathway (Resource Description Framework identification: http://www.reactome.org/biopax/48887Pathway991).

#### 3.1.2 From KEGG enzyme

We also extracted metabolic isoenzyme information from KEGG Enzyme database using the provider’s Representational-state-transfer (REST)-based web service. For this, we first obtained all metabolic enzymes, identified by their corresponding EC numbers, registered in KEGG Enzyme (http://rest.kegg.jp/list/ec). Then, for each enzyme, we obtained all human genes that are associated with the enzyme and created groups of isoenzymes using their gene symbols. For later reference, we keep the primary name of the enzyme and the text-based description of the reaction associated with the corresponding isoenzyme group.

#### 3.1.3 Combining isoenzyme data form multiple resources and filtering

After collecting isoenzyme groups, we pooled isoenzyme groups from these multiple resources. For isoenzyme gene sets that came from different resources but had the exact gene composition, we used the following priority for the data resources to decide which copy to keep in the final analysis: (i) KEGG Enzyme, (ii) Reactome (Croft *et al.*, 2011) and (iii) HumanCyc (Romero *et al.*, 2005).

### 3.2 Collecting drug–target data

To collect drug–target data from multiple resources, we used PiHelper and aggregated data from all data resource it supports by default: (i) DrugBank (Knox *et al.*, 2011), (ii) KEGG Drug (Kanehisa *et al.*, 2012), (iii) Rask-Andersen *et al.*, 2011, (iv) Genomics of Drug Sensitivity in Cancer (Yang *et al.*, 2013), (v) Garnett *et al.*, 2012 and (vi) Cancer.gov (http://cancer.gov).

We ran PiHelper with the default parameters and exported all aggregated drug–target data in TSV (tab-separated values) format as previously described (Aksoy *et al.*, 2013). This provided us with a list of genes that can be targeted with a drug, and we used this information to annotate all genes in our isoenzyme gene sets.

### 3.3 Labeling genes using additional annotations

#### 3.3.1 Annotating tissue-specific expression patterns

For tissue-specific gene expression annotation, we used data produced by TiGER (Liu *et al.*, 2008). We downloaded raw file containing tissue-specific UniGene lists and mapped this information, i.e. whether the expression of a gene is restricted to a single tissue, using gene symbol to UniGene maps from the same provider. We also adopted the tissue terminology used by TiGER and annotated cancer studies we used in our study in accordance with this terminology (Table 1).

#### 3.3.2 Annotating essential genes

To annotate genes that are known to be essential in a model organism, we used data provided by DEG (Zhang *et al.*, 2004). For this, we downloaded the whole database and used gene symbol-based annotations for only eukaryotes. We annotate all human genes in the database as essential in our analysis. For non-human essential genes, we used homology-group datasets provided by the HomoloGene (http://www.ncbi.nlm.nih.gov/homologene) to map these genes to their human homologues. When annotating a gene as essential, we always include the species information as part of the annotation for future reference. We collected annotations from the following model organisms: (i) *Homo sapiens*, (ii) *Mus musculus*, (iii) *Drosophila melanogaster*, (iv) *Saccharomyces cerevisiae*, (v) *Caenorhabditis elegans*, (vi) *Danio rerio* and (vii) *Arabidopsis thaliana*.

### 3.4 Handling cancer studies and genomic profiles

We accessed public data for cancer studies listed in Table 1 using cBioPortal’s web service [(Cerami *et al.*, 2012; Gao *et al.*, 2013); http://cbioportal.org]. For each study, (i) we first collected all case IDs, (ii) we then obtained categorized gene-centric CNA data (when possible, we used data generated through either GISTIC or RAE algorithms: −2: homozygous deletion; −1: heterozygous deletion; 0: diploid; 1: gain; 2: amplification) (Mermel *et al.*, 2011; Taylor *et al.*, 2008), (iii) when available, we used normalized Z-scores for gene-centric mRNA expression and treated values <−2 as underexpressed for a particular sample and (iv) manually assigned tissues based on the type of the cancer.

One exception to these general rules was the Cancer Cell Line Encyclopedia (CCLE), where normalized mRNA expression data was missing. For this study, we used median-normalized gene-centric probe levels and treated log2 values <5, which corresponds to upper limit of the lower quartile of all expression data, as underexpressed.

A list of genomic profile IDs that were used for this analysis can be found within the Supplementary Material. Further details for each genomic profile can be accessed from the cBioPortal Web site: http://cbioportal.org.

## 4 DISCUSSION

Cancer cell contains many somatic genomic alterations, some of which may result in therapeutic vulnerabilities. Therapeutic approaches targeting such vulnerabilities are promising because they are expected to be lethal to cancer cells but not to healthy (e.g. non-cancer) cells, thus reducing the potential for toxic side effects. Here we present a systematic approach to identify a subset of such vulnerabilities, involving metabolic pathways, by taking advantage of publicly available data resources. As a proof of concept, we ran our analysis on 16 cancer studies available via the cBioPortal for Cancer Genomics and predicted 4104 metabolic vulnerabilities. We included the CCLE in our analysis as a separate cancer study, and this allowed us to match vulnerabilities in tumor samples with those in cell lines. Overall, we found 2706 vulnerabilities resulting from 220 distinct homozygous deletion events in 1019 tumor samples. In all, 71% of these vulnerability-causing homozygous deletions were also present in at least one cell line, therefore, opening the possibility of testing a majority of these predicted vulnerabilities *in vitro*. Reassuringly, using this systematic method, we were able to detect a previously verified metabolic vulnerability, which is due to a homozygous deletion affecting an enolase isoenzyme (Muller *et al.*, 2012) (Supplementary Fig. S2). Unlike other studies that have previously predicted metabolic vulnerabilities using a theoretical model of cancer metabolism, here we interpreted all datasets in a sample-specific manner (Folger *et al.*, 2011). This helped us capture many vulnerabilities that were not reported previously (Table 2) (Folger *et al.*, 2011; Muller *et al.*, 2012).

Furthermore, we based our analysis on homozygous deletions in cancer samples with a particular focus on metabolic pathways, but our method can easily be extended to signaling pathways and also to any disabling genomic or epigenomic event, such as mutations and hyper-methylation events. We restricted our analysis to consider only homozygous deletions because at the time of the study, the number of samples that have a copy-number profile was considerably higher compared with the number of samples that have either mutation or methylation profile. Moreover, we only used metabolic pathways because details of metabolic reactions are provided at a better level of granularity in many of the pathway data resources. This allowed us to infer potentially synthetic lethal gene sets from the pathway resources with higher confidence. For many signaling pathways, this type of inference is considerably harder to accomplish because they are not as well characterized and well curated as metabolic pathways yet.

The quality of our vulnerability predictions highly depends on the quality of the homozygous deletion calls made for each metabolic gene. A false-positive homozygous deletion call, for example, will also lead to a false-positive vulnerability prediction in our analysis. To overcome this problem, we assign a higher score to vulnerabilities when the homozygously deleted gene is also underexpressed in a specific sample. Another likely source of false-positive predictions is our assumption that all metabolic reactions are essential for cell viability, and therefore, genes catalyzing the same reaction form a synthetic lethal group. These types of issues, however, can be easily addressed by testing a predicted vulnerability *in vitro* using one of the cell lines that has the vulnerability of interest.

To better prioritize the vulnerabilities in terms of their applicability to the clinic and their reliability, we assigned a score (over 4.0) to each individual vulnerability we identified based on the following criteria. First, to emphasize the likelihood of homozygous deletion being true, we checked whether transcripts of homozygously deleted genes are also expressed relatively at low levels compared with the diploid samples. Next, we looked whether the suggested drug to exploit a vulnerability is either FDA-approved or already being used in cancer therapy, where satisfying either criteria indicates not only better availability of the drug for validation experiments but also relatively easier translation to clinical trials. Finally, we checked whether targeting the vulnerability will inhibit an essential gene, hence increasing the possibility of a toxic effect for the host.

These criteria reflect a subjective view of a reliable vulnerability prediction and can be expanded by incorporating more annotation and supportive datasets to the analysis. For example, various drug screen studies and small hairpin RNA knockdown assays provide relative sensitivities of cell lines toward inhibition of various cellular species as public datasets (Barretina *et al.*, 2012; Cheung *et al.*, 2011), and this information can be further used in the context of vulnerabilities, where sensitivities that can be explained by a predicted vulnerability are given an extra score. Another possible extension to our scoring scheme is to give extra scores to vulnerabilities for which suggested drugs are currently being tested in clinical trials for the tumor type that matches the patient’s.

Our analysis identifies only vulnerabilities for which the target gene can selectively be inhibited by a compound, but for each vulnerability prediction, we also report drugs that are less selective and yet potentially interesting for exploiting a vulnerability. Considering both selective and non-selective drugs, our results show that 44% of the identified vulnerabilities can potentially be targeted with an FDA-approved drug; moreover, a smaller fraction, 8%, of all vulnerabilities seems to be targetable with drugs that are both FDA-approved and already being used in cancer therapy (Table 3).

**Fig. 5.**
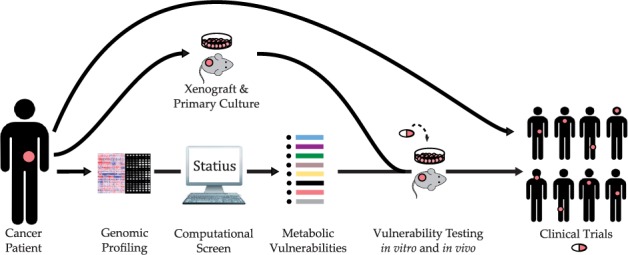
Potential applications to personalized and/or precision cancer therapy. Our method can easily be extended to identify vulnerabilities from the genomic profile of a recently diagnosed cancer patient. The candidate vulnerabilities for this patient can then be tested on primary cell cultures or xenograft models (established from patient’s tumor sample) with drugs of interest as suggested by our analysis. Once the vulnerability is verified, ‘basket’ clinical trials can be designed to test the efficacy of the drug on patients who are predicted to have this particular vulnerability

Opportunities to exploit these vulnerabilities have previously been overlooked because genomic alterations that cause such vulnerabilities are relatively less frequent within each cancer study. We show that with the help of a systematic method that can efficiently combine data from diverse resources, it is possible to identify vulnerabilities that cover a considerable number of patients when aggregated across different cancer studies. We believe this type of systematic and patient-specific treatment suggestion will prove essential especially in designing ‘basket trials’ that will investigate the effects of a targeted agent against a specific genetic alteration (Fig. 5).

## Supplementary Material

Supplementary Data
