# Supplementary material for: Prediction of individualized therapeutic vulnerabilities in cancer from genomic profiles
Source: Bioinformatics. 2014 Mar 24;30(14):2051–9. doi: 10.1093/bioinformatics/btu164 (PMC4080742; doi:10.1093/bioinformatics/btu164)
Supplement: Supplementary Data [file supp_30_14_2051__index.html]

Prediction of individualized therapeutic vulnerabilities in cancer from genomic profiles — Prediction of individualized therapeutic vulnerabilities in cancer from genomic profiles — Prediction of individualized therapeutic vulnerabilities in cancer from genomic profiles — Supplementary Data 

# Prediction of individualized therapeutic vulnerabilities in cancer from genomic profiles

## Supplementary Data

files

**Files in this Data Supplement:**

- Supplementary Data - pdf file
- Supplementary Data - pdf file
- Supplementary Data - tsv file
