# Supplementary material for: Prediction of individualized therapeutic vulnerabilities in cancer from genomic profiles
Source: Bioinformatics. 2014 Mar 24;30(14):2051–9. doi: 10.1093/bioinformatics/btu164 (PMC4080742; doi:10.1093/bioinformatics/btu164)
Supplement: Supplementary Data [file supp_btu164_S1_SUPPLEMENTARY_MATERIALS.pdf]

S1 SUPPLEMENTARY MATERIALS

This document contains supplementary material for "Prediction of individualized therapeutic vulnerabilities in cancer from genomic profiles" (Aksoy et al., Bioinformatics 2014).

Table S1. The 20 most common candidate therapeutic vulnerabilities detected in the analysis of 5971 cancer samples from 16 different studies. Our analysis revealed a total of 263 candidate vulnerabilities.

Each of these vulnerabilities is associated with a gene set that represents isoenzymes that catalyze a metabolic reaction and deletion of one or more partner genes results in a vulnerability if there are targeted drug(s) that can selectively inhibit the other enzymes in the gene set. The majority of the vulnerabilities in tumors were also present in at least one cell line. Using Cancer Census Gene data set (http://bit.ly/sangercancergenes), we identified the closest cancer gene(s) to the homozygously deleted gene for each of these vulnerabilities.

#	Isoenzyme set	Deleted gene	Nearest cancer genes	Tumors	Cell lines	Metabolic reaction	Drugs
1	EXTL2, EXTL3	EXTL3	8p21.1 - HOOK3, WHSC1L1, WRN, PCM1	173	47	glucuronyl-galactosyl-proteoglycan 4-alpha-N-acetylglucosaminyltransferase	Uridine-Diphosphate-N-Acetylglucosamine
2	PAPSS1, PAPSS2	PAPSS2	10q23.2 - FAM22A, PTEN, NFKB2, TLX1	97	17	adenylyl-sulfate kinase	Adenosine-5'-Phosphosulfate
3	CPT1C, CPT1B, CPT2, CPT1A	CPT1B	22q13.33 - MKL1, MN1, EP300, MYH9	90	10	carnitine O-palmitoyltransferase	L-Carnitine
4	A2M, BMP1	BMP1	8p21.3 - HOOK3, WHSC1L1, WRN, PCM1	68	2	HDL-mediated lipid transport	Becaplermin
5	GOT1, GOT2, GOT1L1	GOT1L1	8p11.23 - RUNXBP2, FGFR1, HOOK3, WHSC1L1	65	27	aspartate degradation II	Maleic acid, 4'-Deoxy-4'-Acetylyamino-Pyridoxal-5'-Phosphate
6	GYG1, GYG2	GYG2	Xp22.33 - CRLF2, P2RY8	58	0	glycogenin glucosyltransferase	UDP-D-galactose
7	ATP2C1, ATP2C2	ATP2C2	16q24.1 - CDH1, CDH11, CBFA2T3, FANCA	57	20	calcium transport I	Desflurane/Halothane
8	ADA, ADAT3	ADAT3	19p13.3 - GNA11, MAP2K2, STK11, MLLT1, TCF3	53	13	adenine and adenosine salvage III	Pentostatin
9	SAT1, SAT2	SAT2	17p13.1 - RAB5EP, TP53, USP6, PER1, YWHAE	48	44	diamine N-acetyltransferase	Diminazene
10	FNTA, PGGT1B	PGGT1B	5q22.3 - APC	47	15	protein geranylgeranyltransferase type I	Tipifarnib
11	DHFR, DHFRL1	DHFR	5q14.1 - PIK3R1, APC	47	5	dihydrofolate reductase	5-Chloryl-2,4,6-Quinazolinetriamine
12	AKR1B10, AKR1B1, CYP2E1	CYP2E1	10q26.3 - FGFR2	42	33	methylglyoxal degradation III	Tolrestat
13	TK1, TK2	TK2	16q21 - CBFB, MAF, CDH1, CDH11	42	8	thymidine kinase	Dithioerythritol
14	ACAT1, ACAT2	ACAT2	6q25.3 - EZR	39	23	acetyl-CoA C-acetyltransferase	Sulfasalazine
15	ENO1, ENO2, ENO3	ENO1	12p13.31 - CCND2, ETV6, ZNF384, ELKS	37	18	phosphopyruvate hydratase	2-Phosphoglycolic Acid
16	ACAT1, ACAT2	ACAT1	11q22.3 - BIRC3, MAML2, DDX10, ATM	36	22	acetyl-CoA C-acetyltransferase	Pyripyropene A
17	MTHFD1, MTHFD1L	MTHFD1L	6q25.1 - ECT2L, EZR	34	24	formate—tetrahydrofolate ligase	LY374571/LY249543
18	ALDH2, ALDH3A2	ALDH3A2	17p11.2 - BHD, HCMOGT-1, MAP2K4	30	28	putrescine degradation III	Daidzin
19	TRYP1, CAT	TYRP1	9p23 - MLLT3, PSIP2, CD274, JAK2	12	71	ethanol degradation IV	Fomepizole
20	AMY1A/B/C, AMY2A, AMY2B	AMY1A/B/C	1p21.1 - NOTCH2, FUBP1, NRAS, BCL10	1	61	alpha-amylase	Acarbose

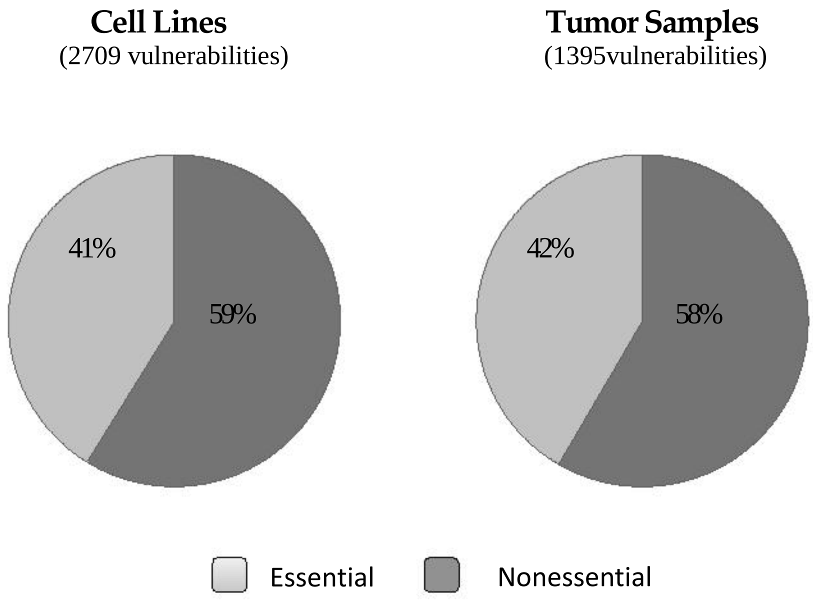

Figure S1. 1695 out of 4104 (41%) vulnerabilities that we identified, intervention with drugs will involve targeting at least one essential enzyme (see Methods for more details on annotating genes as essential). These vulnerabilities correspond to 41% of the vulnerabilities in cell lines and 42% in tumor samples

ENO1-deletion and vulnerability statistics

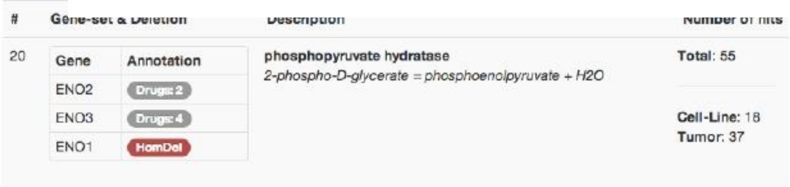

#	Gene-set & Deletion		Description	Number of hits
20	Gene	Annotation	**phosphopyruvate hydratase** *2-phospho-D-glycerate = phosphoenolpyruvate + H2O*	**Total**: 55
	ENO2	Drugs: 2		
	ENO3	Drugs: 4		**Cell-Line**: 18 **Tumor**: 37
	ENO1	HomDel		

(a)

Pathway representation

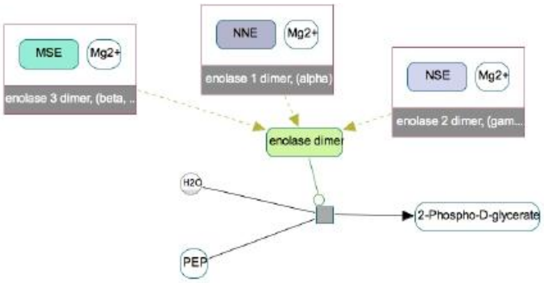

Vulnerabilities in GBM

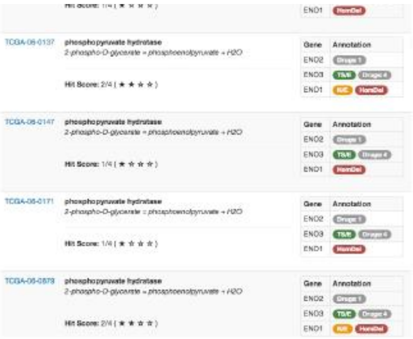

(b) (c)

Figure S2. Details of the enolase vulnerability. (a) We found 37 tumor samples and 18 cell lines that have the ENO1 homozygous deletion, which was previously shown to cause a vulnerability in glioblastoma samples (2). (b) Enolase dimer (ENO1/2/3/) catalyzes (green arrow) the metabolic reaction (gray square box) that converts PEP and H_2O into 2-phospho-D-glycerate (connected to the reaction with black lines). The reaction was visualized by ChiBE (32).
(c) Five of the TCGA Glioblastoma cases that have ENO1-deletion (HomDel; red) and therefore are predicted to have a vulnerability in the enolase reaction context. In two of these cases, ENO1 also seems to be under-expressed (N/E; orange) compared to the glioblastoma cohort. ENO3 is known to be expressed only in muscle tissues, hence is not expected to be expressed (TS/E; green) in these glioblastoma samples.
